# Exploiting the anticancer effects of a nitrogen bisphosphonate nanomedicine for glioblastoma multiforme

**DOI:** 10.1186/s12951-021-00856-x

**Published:** 2021-05-04

**Authors:** Lynn N Jena, Lindsey A Bennie, Emma M McErlean, Sreekanth Pentlavalli, Kim Glass, James F Burrows, Vicky L Kett, Niamh E Buckley, Jonathan A Coulter, Nicholas J Dunne, Helen O McCarthy

**Affiliations:** 1 School of Pharmacy, Queen’s University Belfast, 97 Lisburn Road, Belfast, BT9 7BL UK; 2 School of Mechanical and Manufacturing Engineering, Dublin City University, Dublin 9, Ireland; 3 Centre for Medical Engineering Research, School of Mechanical and Manufacturing Engineering, Dublin City University, Dublin 9, Ireland; 4 Department of Mechanical and Manufacturing Engineering, School of Engineering, Trinity College Dublin, Dublin 2, Ireland; 5 Advanced Manufacturing Research Centre (I-Form), School of Mechanical and Manufacturing Engineering, Dublin City University, Glasnevin, Dublin 9, Ireland; 6 Advanced Materials and Bioengineering Research Centre (AMBER), Royal College of Surgeons in Ireland and Trinity College Dublin, Dublin, Ireland; 7 Advanced Processing Technology Research Centre, Dublin City University, Dublin 9, Ireland; 8 Trinity Centre for Biomedical Engineering, Trinity Biomedical Sciences Institute, Trinity College Dublin, Dublin 2, Ireland; 9 School of Chemical Sciences, Dublin City University, Dublin 9, Ireland

**Keywords:** Glioblastoma Multiforme, Bisphosphonate, Nanoparticles, Anticancer, RALA peptide, Nanomedicine

## Abstract

Glioblastoma multiforme (GBM) is an incurable aggressive brain cancer in which current treatment strategies have demonstrated limited survival benefit. In recent years, nitrogen-containing bisphosphonates (N-BPs) have demonstrated direct anticancer effects in a number of tumour types including GBM. In this study, a nano-formulation with the RALA peptide was used to complex the N-BP, alendronate (ALN) into nanoparticles (NPs) < 200 nm for optimal endocytic uptake. Fluorescently labelled AlexaFluor®647 Risedronate was used as a fluorescent analogue to visualise the intracellular delivery of N-BPs in both LN229 and T98G GBM cells. RALA NPs were effectively taken up by GBM where a dose-dependent response was evidenced with potentiation factors of 14.96 and 13.4 relative to ALN alone after 72 h in LN229 and T98G cells, respectively. Furthermore, RALA/ALN NPs at the IC_50,_ significantly decreased colony formation, induced apoptosis and slowed spheroid growth *in vitro.* In addition, H-Ras membrane localisation was significantly reduced in the RALA/ALN groups compared to ALN or controls, indicative of prenylation inhibition. The RALA/ALN NPs were lyophilised to enhance stability without compromising the physiochemical properties necessary for functionality, highlighting the suitability of the NPs for scale-up and *in vivo* application. Collectively, these data show the significant potential of RALA/ALN NPs as novel therapeutics in the treatment of GBM.

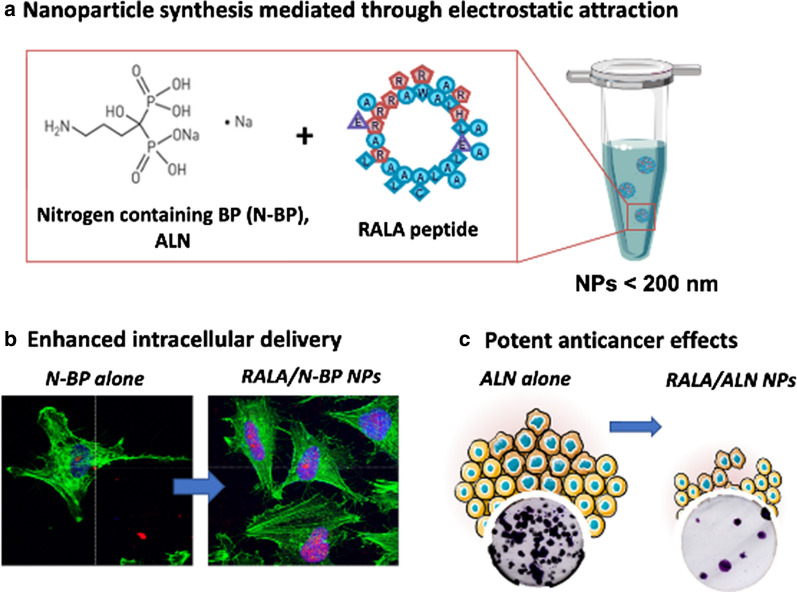

## Introduction

Glioblastoma multiforme (GBM) is the most malignant primary brain tumour, derived from glial cells. Unlike other solid cancers, GBM rarely colonise regions external to the central nervous system (CNS) [1]. Post-diagnosis, median survival remains low at 6.9 months and the 5-year overall survival rate is less than 9.8% despite multimodal therapy [2]. Surgery, radiotherapy plus adjuvant temozolomide chemotherapy represent the standard of care for newly diagnosed GBM patients [3]. Unfortunately, GBM recurs in up to 90% of cases that ultimately leads to patient death [4]. To prolong tumour control and patient survival, novel therapeutic strategies are required.

Clinically, bisphosphonates (BPs) have proven safety and efficacious in the management of bone resorptive diseases such as Paget’s disease, osteoporosis and metastatic bone disease [5]. However, BPs are characterised by low bioavailability with less than 1% of the oral therapeutic dose absorbed due to the high affinity for bone mineral [6]. Approximately 50% of the orally delivered drug is renally excreted and the remainder is concentrated on bone surface [7]. Although generally tolerated, adverse effects can occur due to free unbound BP. These effects typically occur in the upper gastrointestinal (GI) tract with osteonecrosis of the jaw observed following long term use [8]. Subsequently, this limits the clinical usefulness of BPs.

BPs can be divided into two classes where a clear structure-function relationship exists. Each class of BP share the same P-C-P backbone structure where the presence of two phosphonate groups confers high affinity for hydroxyapatite, a major component of bone. This is necessary to inhibit osteoclast activity, with the potency determined by the R1 and R2 side chains, respectively [9]. First generation BPs, also known as non-nitrogen containing BPs, incorporate into adenosine triphosphate (ATP)-containing compounds resulting in the production of an ATP analogue to induce cellular apoptosis [10]. Non-N-BPs are the less potent antiresorptive agents, whereas second and third generation N-BPs exhibit increased potency through the existence of a nitrogen-containing R2 side chain [11]. In addition to hydroxyapatite binding, N-BPs impair intracellular signalling in osteoclasts through the inhibition of the enzyme farnesyl pyrophosphate synthase (FPPS). FPPS is a key enzyme in the mevalonate pathway, involved in osteoclast morphology and the post-translational prenylation of small GTPase signalling proteins, including Ras, Rac, Rho which are all essential for cellular function and survival [12].

Moreover, it is now clear that N-BPs can affect tumour cells through direct and indirect anti-tumour effects in preclinical models. BPs inhibit angiogenesis, induce apoptosis, reduce migration and invasion and subsequently control cancer cell proliferation. BPs have demonstrated *in vitro* anticancer activity against various cancer cell lines, including breast, prostate and brain [5, 11, 13, 14]. Ottewell *et al.* found when zoledronic acid was administered 24 h post-treatment with doxorubicin in MDA-MB-436 cells, this led to an increase in the expression of proapoptotic genes, while the presence of unprenylated Rap1 indicated induced apoptosis [15]. Furthermore, previous research conducted within our group by Massey *et al.,* found that the potency of alendronate (ALN) increased when complexed with the RALA peptide in MDA-MB-231 breast cancer and PC-3 prostate cancer cell lines generating a potentiation factor of 5.7 and 4.1, respectively [5].

McCarthy *et al.* developed RALA, a 30-amino acid, cationic, amphipathic peptide (N-WEARLARALARALARHLARALARALRACEA-C), with a secondary structure that facilitates the complexation of anionic compounds through electrostatic interactions into nanoparticles (NPs) < 200 nm [5, 16–22]. Due to the small size and charge, NPs are internalised *via* endocytic mechanisms [17]. Following endocytosis, NPs are generally held within acidic endosomes. At the low pH of the mature endosome (pH< 5), many drug molecules become degraded, hindering the accumulation of therapeutic concentrations at the active site [23]. However, the pH-responsive fusogenic activity of RALA enables the release of complexed cargo before it can be degraded [17]. As the endosome becomes more acidic, basic amino acid residues in the peptide sequence become protonated which are associated with an increased alpha-helicity. It is proposed that protonation of the histidine amino residue contribute to the ‘proton sponge effect’ leading to increased proton influx. This in turn forces the influx of water and CL^-^ ions into the endosome causing it to swell and eventually rupture [24]. The hydrophobic domain is able associate with the lipid bilayer, causing lysis of the organelle and efficient release of the cargo.

Many BP-containing compounds have been synthesised and show extremely promising inhibitory properties against key enzymes in the mevalonate pathway [14]. However, due to the known limitations of the pharmacokinetics of BPs, complexing these agents with the RALA peptide offers an attractive solution to increase delivery of BPs to non-osseous tumour sites. Studies by Massey *et al.* demonstrates the original BP structure can be retained and through complexation with the RALA peptide, the anticancer effects in prostate and breast cancer cells are potentiated at a lower dose. Furthermore, localised treatment *in vivo* resulted in significant tumour growth delay in a PC-3 prostate xenograft model; a 56.3% increase in survival time of RALA/ALN NP treated Balb-c-SCID mice was observed compared to the untreated control group (P < 0.001) [5].The current study aims to determine the anticancer effects in GBM through a RALA/N-BP formulation which is used for intravenous rather than local administration. RALA facilitates the intracellular delivery of ALN into cancer cells to enhance the therapeutic efficacy and potentially negates adverse effects associated with BP administration. The subsequent lyophilisation of NPs enables the application of higher doses for systemic delivery to the brain. Notably, cationic molecules are transported across the BBB through adsorption-mediated transcytosis which is triggered via electrostatic interactions between positively charged moieties of cationic agent and negatively charged membrane surface domains on the BBB [25].

The unique combination of amphipathicity, cationicity, and pH-responsive fusogenicity present RALA as an exciting candidate for multifunctional drug delivery [26]. For the first time, the nano-formulation of ALN in a RALA NP is interrogated as a potential therapy for GBM.

## Experimental procedures

### Reagents

#### BPs

N-BPs were obtained from commercial sources: ALN monosodium trihydrate (MW: 249.1 g/mol) (Invitrogen, Thermo Fisher Scientific, UK was reconstituted in TE buffer (Invitrogen, Thermo Fisher Scientific, UK) to give a final concentration of 4 mM (1 μg/μL). AlexaFluor®647-Risedronate (AF647-RIS) (MW: 1198 g/mol) (BioVinc, California, USA) was reconstituted in TE buffer to give a final concentration of 1 μg/μL. The reconstituted BPs were stored at room temperature (RT) until further use.

#### RALA

The RALA peptide was synthesised by solid-state synthesis (FMOC chemistry), provided in acetate form at > 95% purity as a lyophilised powder (Biomatik, USA). RALA was reconstituted in UltraPure DNase/RNase-Free Distilled Water (Invitrogen, Thermo Fisher Scientific, UK) and stored in aliquots at – 20 °C until use.

#### Plasmid EGFP-C3-H-Ras

The pEGFP-C3-H-Ras (pEGFP-H-Ras) plasmid was provided by Dr. James Burrows (Queen's University Belfast, UK) [27]. Plasmids were propagated in MAX Efficiency® DH5α™ Competent Cells (Thermo Fisher Scientific, UK) purified using PureLink®HiPure Plasmid Filter Maxiprep Kit (Thermo Fisher Scientific, UK) and quantified by UV absorption at 260 nm.

### Methods

#### Cells

LN229 and T98G glioblastoma cell lines (ATCC, Manassas, VA) were maintained in Dulbecco’s modified eagle’s medium (DMEM) (Invitrogen, Thermo Fisher Scientific, UK) supplemented with 10% foetal bovine serum (FBS) (Invitrogen, Thermo Fisher Scientific, UK). All cell lines were authenticated by short tandem repeat (STR) profiling carried out by the suppliers and routine testing revealed that these cells were Mycoplasma-free. All experiments were conducted at 37°C in a humidified atmosphere of 5% CO_2_/95% O_2_.

#### Formulation of RALA/ALN NPs

Each BP (1 μg or 0.1 μg AF647-RIS) was complexed with the RALA peptide (1 μg/μL) at a range of mass ratios. For example, to achieve a ratio of 10:1, 10 μg of RALA peptide was added to 1 μg of BP dissolved in TE buffer and the total volume was adjusted to 50 μL with TE buffer (Invitrogen, Thermo Fisher Scientific, UK) pH 8.0 and UltraPure DNase/RNase-Free Distilled Water (Invitrogen, Thermo Fisher Scientific, UK). For RALA/AF647-RIS NPs, the pH was adjusted to 7.0. Samples were left to incubate at RT for 30 min before physiochemical characterisation and functional studies commenced.

#### Particle size and zeta potential analysis

The mean hydrodynamic size of formulated NPs was measured by dynamic light scattering (DLS) using a Nano ZS Zetasizer instrument with DTS software (Malvern Instruments, UK). Following size measurement, UltraPure DNase/RNase-Free Distilled Water (Invitrogen, Thermo Fisher Scientific, UK) at a volume of 1 mL was used to dilute 50 μL of the NP sample. The sample was placed into a folded capillary zeta cell (Malvern Instruments, UK) to allow measurement of the zeta potential, detected through Laser Doppler Velocity. All measurements were conducted at 20 °C.

#### Temperature analysis

To assess NP stability in cold storage, room temperature and body temperature, particle size was measured over a range of temperatures. RALA/ALN NPs were prepared at mass ratio 10:1 and incubated at RT for 30 min to allow complexation. The Nano ZS Zetasizer instrument with DTS software (Malvern Instruments, UK) was used to measure the mean hydrodynamic size from 4–40 °C at 3 °C intervals. Samples were allowed to equilibrate at each temperature for 120 s prior to measurement.

#### Incubation study

To assess the physical nanoparticle stability over a period of time, RALA/ALN NPs were prepared at mass ratio 10:1 and incubated at RT over a period of 28 days; the mean hydrodynamic size was measured daily using the Nano ZS Zeta sizer and DTS software (Malvern Instruments, UK).

#### Lyophilisation of RALA/ALN NPs

Freeze-drying was performed in the final step of the formulation process to enable long-term storage and to produce concentrated particles*.* RALA/ALN NPs were freshly prepared at mass ratio of 10:1 in a 150 µL volume. After a 30 min incubation period, a 20% w/v trehalose solution (Sigma-Aldrich, UK) was filtered through a 0.45 µm filter and added to NP formulations to give a final concentration of 1–5% w/v. Each NP formula was lyophilised in a 2-mL glass vial in a BenchTop Freeze Dryer (SP Scientific VirTis AdVantage with Wizard 2.0 controller, USA) to form lyophilised RALA/ALN (L-RALA/ALN) NPs. The formulation was slowly frozen at a shelf temperature of − 40 °C for 1 h. Drying was performed at – 35 °C and 120 mTorr for 3 h, − 30° C and 190 mTorr for 4 h, − 25 °C and 190 mTorr for 3 h and 20 °C and 190 mTorr for 8 h. Thereafter, the shelf temperature remained at 20 °C and the pressure was reduced to 50 mTorr for 10 h in the secondary drying step. Lyophilised NPs were stored at RT prior to characterisation and performance evaluation. Immediately prior to use, samples were reconstituted in a 150–200 µL volume of UltraPure DNase/RNase-Free Distilled Water (Invitrogen, Thermo Fisher Scientific, UK).

#### Transmission electron microscopy

RALA/ALN NPs were prepared as described in section “Formulation of RALA/ALN NPs” at mass ratio 10:1 and loaded onto carbon reinforced 400 mesh copper grids (TAAB Laboratories, UK). NPs were left to adhere for 1 h before drying overnight. The grids were subsequently stained with 5% uranyl acetate in methanol for 5 min at room temperature before further drying of the grid. Grids were imaged using a JEM-1400Plus Transmission Electron Microscope (JEOL Inc., USA) at an accelerating voltage of 120 kV to provide high resolution images.

#### Encapsulation efficiency of RALA/AF647-RIS NPs

RALA/AF647-RIS NPs were formulated through complexation of 0.1 μg AF647-RIS across a range of mass ratios (1–20) as described in section “Formulation of RALA/ALN NPs”. NPs were made up to a 50 μL volume and subsequently loaded onto a black 96-well plate in triplicate. Sample fluorescence was analysed by excitation at 648 nm and the fluorescence emission intensity measured at 666 nm using the Cytation™5 Cell imaging Multi-Mode reader (Biotek, USA). The fluorescence intensity of the AF647-RIS only control was taken as 100% fluorescence and 0% encapsulation, therefore, any detected fluorescence of samples was taken as un-encapsulated AF647-RIS. The percentage of un-encapsulated AF647-RIS was used to determine the percentage of encapsulated AF647-RIS.

#### Intracellular uptake of fluorescently labelled BP

Cells were seeded onto a 96-well plate at 1.5 × 10^4^ cells per well and allowed to adhere overnight at 37 °C with 5% CO_2_. Medium was replaced with OptiMEM (Invitrogen, Thermo Fisher Scientific, UK) 2 h prior to treatment. Cells were treated with RALA/AF647-RIS NPs prepared over a range of mass ratios containing 0.1 μg AF647-RIS. NPs were prepared as described in section “Formulation of RALA/ALN NPs” in a total volume of 50 μL and stored under dark conditions. Cells were incubated with NPs for 2 h before media was removed. Thereafter, cells were washed three times with warmed phosphate buffered saline (PBS) and 0.1% trypsin was used to detach cells. Complete media was added, and the cells were centrifuged at 2300 g for 10 min. Subsequently, cells were resuspended in PBS before analysis using the Accuri C6 Plus Flow Cytometer (BD Bioscience, UK).

#### Confocal microscopy

Cells were seeded onto 24-well culture multi-dish plates on 13 mm coverslips (Agar Scientific, UK) at 3 × 10^4^ cells/well and allowed to adhere overnight at 37 °C with 5% CO_2_. Medium was replaced with OptiMEM (Invitrogen, Thermo Fisher Scientific, UK) 2 h prior to treatment. Cells were treated with RALA/AF647-RIS NPs at a mass ratio 10:1 containing 0.1 μg AF647-RIS. NPs were prepared as described in section “Formulation of RALA/ALN NPs”. in a total volume of 50 μL and stored under dark conditions. Cells were incubated with NPs for 2 h, before media was removed. Following treatment, cells were washed three times with warmed PBS, fixed with 4% formaldehyde and permeabilised using 0.1% Triton X -100 (Sigma-Aldrich, UK). The cytoskeleton was stained with AlexaFluor®488-phalloidin (Invitrogen, Thermo Fisher Scientific, UK) for 15 min at RT. Following staining, cells were washed with warmed PBS and mounted on microscope slides (Agar Scientific, UK) with Fluoroshield mounting medium containing DAPI nuclear stain (Invitrogen, Thermo Fisher Scientific, UK). Slides were imaged using a TCS SP8-Leica Microsystems confocal microscope (Leica, UK). Images were analysed using LAS AF Lite Software (Leica, UK).

#### Dose response study

Cell viability was evaluated using the MTS assay with CellTiter 96 Aqueous One Solution Cell Proliferation Assay (Promega, U.K.). LN229 and T98G cells were seeded onto a 96 well plate at 1.5 × 10^4^ cells per well and allowed to adhere overnight at 37 °C with 5% CO_2_. Medium was replaced with OptiMEM (Invitrogen, Thermo Fisher Scientific, UK) 2 h prior to treatment with solutions of BP to achieve a final exposure concentration of 25 μM to 1 mM. RALA/ALN NPs were prepared using a mass ratio of 10:1, such that the final concentration of BP per well was in the range 5 to 75 μM. Cells were incubated for 6 h following treatment before medium was replaced with complete media and subsequently incubated for a further 72 h. Cell viability was assessed according to the manufacturers’ protocol where reagent was added to each well to a final concentration of 10% and plates were returned to the incubator for 2 h. Following incubation, the absorbance value (OD value) was recorded at a wavelength of 490 nm using a FLUOstar Omega microplate reader with MARS Data Analysis software (BMG Labtech, UK), and any background absorbance was subtracted from readings. Cell viability was expressed as a percentage of that of the untreated control where the untreated control is considered to be 100% viable. Data were normalized using GraphPad Prism version 8.1.2. (GraphPad Software, USA), (using the equation Y=100/(1+10^((LogIC_50_-X)*HillSlope))) to recalculate Y values). Nonlinear regression analysis was used to fit log (inhibitor) vs. normalised response -variable slope to the data and the IC_50_ was calculated.

#### Apoptosis analysis

The apoptosis of LN229 and T98G cells was assessed by flow cytometry, using double staining with Annexin V-FITC and propidium iodide (PI) (FITC Annexin V Apoptosis Detection Kit I, BD Biosciences Pharmingen™, USA) as per manufacturers’ guidelines. Prior to staining, cells were treated for 6 h with ALN alone or RALA/ALN NPs at the IC_50._ Cells were harvested from 24-well culture plates with 0.1% trypsin–EDTA solution (Invitrogen, Thermo Fisher Scientific, UK) and washed with PBS. A 100 µl volume of suspended cells was stained with 5 µL Annexin V-FITC and 5 µL PI for 15 min and incubated under dark conditions at RT, before the addition of 400 µL assay buffer. The respective proportions of apoptotic cells and necrotic/late-apoptotic cells were measured on 10,000 cells with an Accuri C6 Plus (BD Bioscience, UK) flow cytometer. Analysis was performed using BD Accuri C6 software (BD Bioscience, UK).

#### Clonogenic assay

LN299 and T98G cell lines were seeded onto 6-well culture plates at 3 × 10^5^ cells/well for 24 h and allowed to adhere overnight. Medium was replaced with OptiMEM (Invitrogen, Thermo Fisher Scientific, UK) 2 h prior to treatment. Cells were treated for 6 h with RALA/ALN NPs (mass ratio 10:1) at the IC_50_ concentration observed from experiments conducted in section “Dose response study”. Following treatment, cells were harvested from 6-well culture plates with 0.1% trypsin–EDTA solution (Invitrogen, Thermo Fisher Scientific, UK) and washed with PBS before counting using a Coulter counter (Beckman-Coulter, ROI). Cells were plated at predetermined cell densities in 6-well plates before incubation for 14 days. Colonies were fixed with 0.4% crystal violet in 70% methanol and were manually counted using a > 50-cell exclusion criteria. Plating efficiency was calculated as the ratio of colonies to cells seeded. The surviving fraction (SF) could then be calculated as the plating efficiency of the treated groups divided by the plating efficiency of untreated cells.

#### Prenylation study

LN229 and T98G cells were seeded onto LabTek II, CC2-treated 4-well chamber slides (Nalge Nunc) at a seeding density of 3 × 10^4^ cells/well. Cells were allowed to adhere overnight at 37 °C with 5% CO_2_. Medium was replaced with OptiMEM (Invitrogen, Thermo Fisher Scientific, UK) 2 h before cells were transfected for 4 h with 0.5 μg/μL pEGFP-H-Ras using the RALA peptide delivery system at N:P 10 [17]. After 24 h, cells were treated with RALA/ALN NPs at mass ratio 10:1 for 6 h and then incubated for a further 72 h prior to fixation with 4% formaldehyde. Slides were sealed with a coverslip and Fluoroshield mounting medium containing DAPI nuclear stain (Thermo Fisher Scientific, UK). Slides were imaged using a TCS SP8-Leica Microsystems confocal microscope (Leica, UK) with a 63x oil objective lens, 1024 × 1024 frame and 400 Hz scanning speed. Images were analysed using LAS AF Lite Software (Leica, UK) and Fiji ImageJ (National Institute of Health, USA).

#### 3D Spheroid culture growth

Spheroid formation is based on cell propensity to self-aggregate when cultured in non-adhesive conditions [28]. LN229 and T98G cells were seeded into Corning® ultra-low attachment clear round bottom 96-well plates (Thermo Fisher Scientific, UK) with 800 cells/well in the presence of 200 μL complete media. Cells were incubated at 37 °C, 5% CO_2_ atmosphere for 48 h to allow for spheroid culture formation prior to analysis. Spheroid measurement was initiated 72 h post-seeding and conducted using the Cell3iMager X neo (SCREEN, Shimadzu Japan). On Day 3, cells were treated with ALN and RALA/ALN NPs. Spheres were measured daily over a 10-day period with cells maintained at 37 °C, 5% CO_2_ atmosphere throughout.

#### Statistical analysis

Control and treatment groups were statistically compared using Student's t-test, one-way or two-way analysis of variance followed by Tukey's multiple comparison test. A p-value of ≤ 0.05 was considered as significant. All values are expressed as the mean ± standard error of the mean of three experiments. Statistical analysis was performed using GraphPad Prism® 8.1.2 (GraphPad Software, USA).

#### Results

### ALN should be formulated at a pH of 8.0 for RALA complexation

**Fig. 1:**
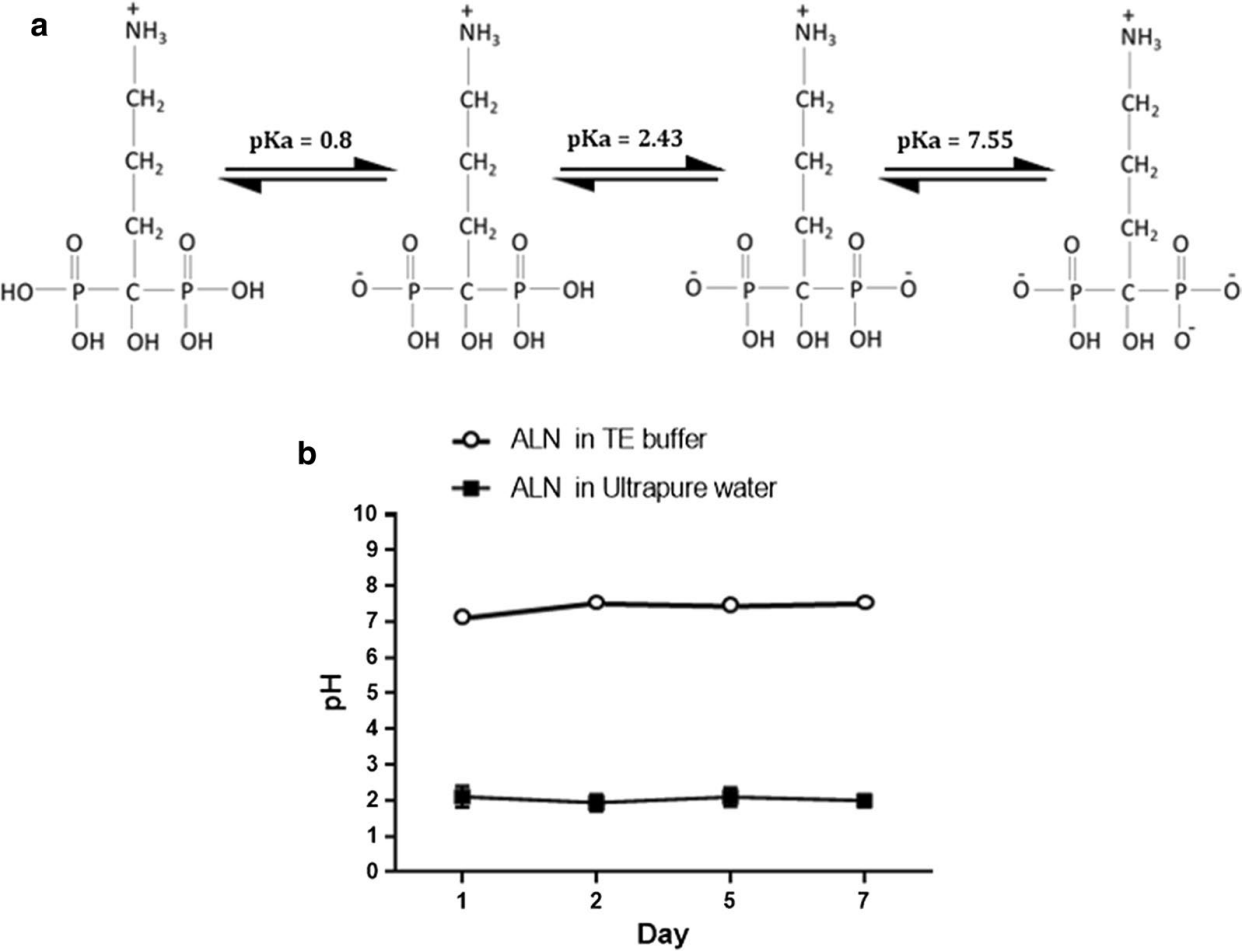
Formulation of ALN monosodium trihydrate stock solution. **a** Dissociation process of ALN sodium. **b** pH of solution of ALN dissolved in UltraPure DNase/RNase-Free Distilled Water and TE buffer observed over a 7-day period at RT. Results are displayed as mean ± SEM, n=3

ALN sodium has four dissociation constants due to the presence of four hydroxyl groups. Dissociation constants and pKa values are regarded as vital physicochemical parameters which impact drug acidity, solubility, metabolism and biological uptake [29]. In particular, pKa values are important for estimating the charged state of polyprotic acids and bases, such as ALN, to determine the existing forms under different pH conditions [29]. When ALN monosodium trihydrate was dissolved in an aqueous solution (pH 7.4), the resultant acidic pH prevented efficient complexation with the cationic peptide due to the pH-responsive nature of RALA (Fig. 1). However, upon increasing pH, the hydroxy groups become deprotonated, exposing an O- group which contributes to the overall negative surface potential of the BP. This phenomenon facilitates electrostatic attraction of the negatively charged BP with the RALA peptide, enabling NP formation. Consequently, RALA/N-BPs NPs were prepared according to a mass ratio of RALA to BP using ALN monosodium trihydrate dissolved in TE buffer (pH 8.0) prior to NP formulation, to achieve an optimal electrochemical state for effective RALA complexation. Nanoformulations remained at a pH ≥ 7 over a period of 7 days.

### RALA complexed ALN forms stable nanosized particles with a positive zeta potential

**Fig. 2:**
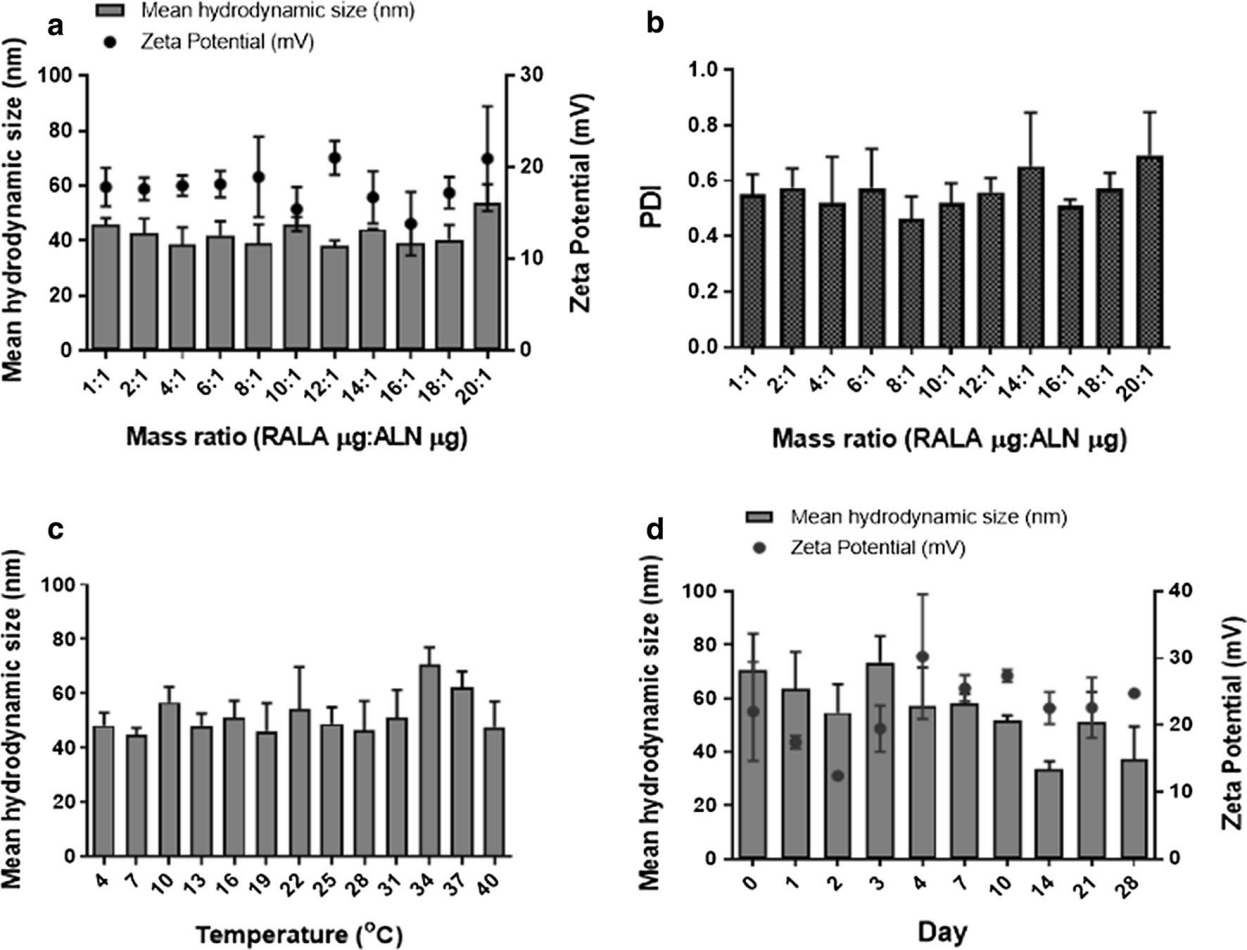
Characterisation of RALA/ALN NPs.** a** Mean hydrodynamic size and zeta potential of RALA/ALN NPs. NPs were formulated at a range of mass ratios and measured using the Malvern Zetasizer Nano ZS instrument at 20 °C through DLS and laser doppler electrophoresis, respectively. **b** The sample quality of RALA/ALN NPs was measured through corresponding polydispersity index (PDI). **c** The mean hydrodynamic size of was used to determine the temperature stability up to 40 °C and the **d** long-stability of RALA/ALN NPs at RT. RALA/ALN NPs were formulated at mass ratio 10 with 1 μg ALN in a 50 μL volume with TE buffer and subsequently left to incubate for 30 min at RT prior to analysis. Results are displayed as mean ± SEM, n = 3

DLS measurements were used to confirm NP formation through the mean hydrodynamic size and zeta potential of RALA/BP NPs. RALA successfully complexed into nanosized particles less than 200 nm at all mass ratios, optimal for cell uptake via endocytosis (Fig. 2a). From mass ratio 1:1 onwards, a zeta potential of ~19 mV was observed with a steady PDI between 0.4 and 0.7. Following initial characterisation, RALA/ALN NPs at mass ratio of 10:1 were selected for further stability analysis. The particle size was analysed over a range of temperatures from 4 to 40 °C with 3 °C intervals (Fig. 2c). Temperature did not significantly impact the particle size of RALA/ALN NPs, with no aggregation observed. NPs remained stable from 4 to 40 °C**,** optimal for refrigerated storage and use at biological temperatures. Moreover, RALA/ALN NPs were examined over a period of time at RT (19 °C) to assess the long-term stability of the formulated NPs (Fig. 2d). Particles exhibited stability up to 28 days in solution as indicated by the mean hydrodynamic size which remained less than 100 nm, with no significant changes in size (*P > 0.05*). A continuous positive zeta potential confirmed RALA was able to effectively complex ALN up to 28 days.

### RALA complexation enhances the cell uptake of fluorescently labelled N-BP

The structure of ALN lacks strong chromophores therefore ultraviolet absorption or fluorescent methods cannot be employed for detection or visualisation [29]. AlexaFluor®647 labelled Risedronate (AF647-RIS) was used as a fluorescent N-BP analogue to determine encapsulation of N-BP with the RALA peptide and to visualise intracellular delivery.

**Fig. 3:**
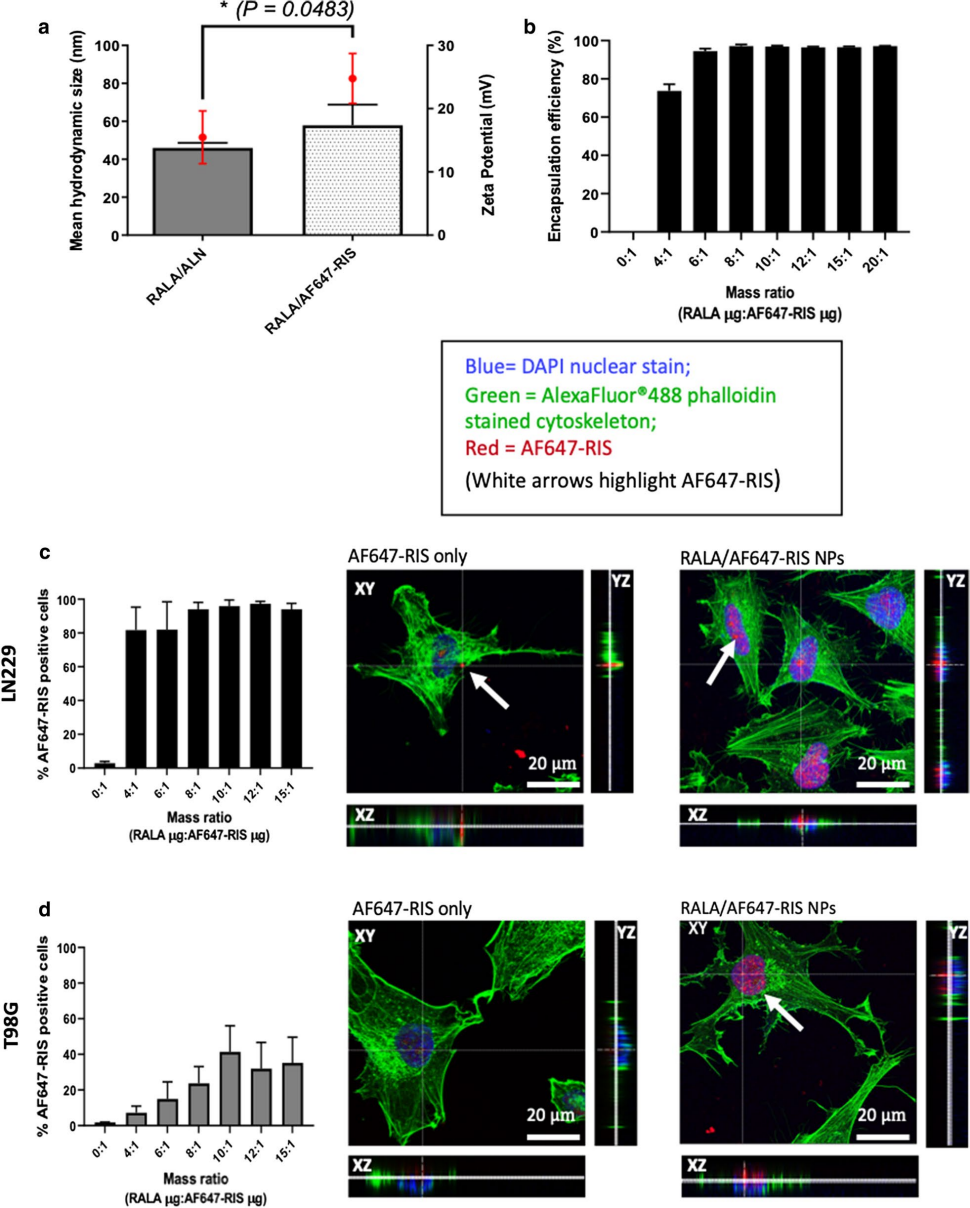
Characterisation and intracellular delivery of RALA/AF647-RIS NPs. **a** Mean hydrodynamic size and zeta potential of RALA/AF647-RIS NPs. Particles were formulated at mass ratio 10 with 1 μg AF647-RIS and were subsequently measured using the Malvern Zetasizer Nano ZS instrument at 20 °C through DLS and laser doppler electrophoresis, respectively. **b **Encapsulation efficiency assay of RALA complexed AF647-RIS NPs at a range of mass ratios. Sample fluorescence was measured at 666 nm using the Cytation™5 Cell imaging Multi-Mode reader (Biotek, USA). The fluorescence intensity of AF647-RIS only control was taken as 100% fluorescence and 0% encapsulation, therefore any detected fluorescence of samples was taken as un-encapsulated fluorescent BP. The percentage of un-encapsulated AF647-RIS was used to determine the percentage of encapsulated AF647-RIS. The intracellular uptake of AF647-RIS NPs in c LN229 and d T98G cells (white arrows denote red fluorescence of AF647-RIS). Particles were prepared over a range of mass ratios containing 0.1 μg AF647-RIS. Cells were subsequently treated with RALA/AF647-RIS NPs for 2 h before flow cytometry. Confocal microscopic images exhibit the intracellular localisation of uncomplexed and RALA complexed AF647-RIS 2 h post-treatment. Slides were imaged using a TCS SP8-Leica Microsystems confocal microscope (Leica, UK) with a 63x oil objective lens and subsequently analysed using LAS AF Lite Software (Leica, UK). Results are reported as mean ± SEM, n=3, and images are representative of three independent repeats. Scale bars = 20 μM

RALA successfully complexed AF647-RIS despite the higher MW, to form positively charged NPs (Fig. 3a). Particles were compared to RALA/ALN NPs formulated at mass ratio 10 and showed a significant increase in overall zeta potential, from + 15.5 to + 24.8 mV with no significant change to mean hydrodynamic size. At mass ratio 4:1, RALA encapsulated 73.7% of AF647-RIS, increasing to > 95% at mass ratios of 6:1 or greater (Fig. 3b).

Flow cytometry determined the intracellular uptake efficiency of RALA/AF647-RIS NPs at varying mass ratios in LN229 and T98G cell lines (Fig. 3c, d). As mass ratio increased, so did the percentage uptake of AF647-RIS in both cell lines. LN229 cells showed the most impressive cell uptake ranging from 80% to 98%, compared to T98G cells where uptake ranged from 7% to 42%. Confocal microscopy was used to determine the intracellular localisation of both RALA-complexed and uncomplexed AF647-RIS, 2 h post treatment in LN229 and T98G cell lines. Cells treated with uncomplexed AF647-RIS exhibited negligible cytoplasmic or nuclear localisation, indicating low cell uptake. This correlates with the flow cytometric analysis where uptake of uncomplexed AF647-RIS reached only 4.9% in the LN229 cell line. In contrast, intracellular accumulation was observed in all cell lines treated with RALA/AF647-RIS NPs (Fig. 3c, d). RALA/AF647-RIS NPs distributed in a close proximity or within the nuclei of cells as evidenced through the orthogonal section.

### RALA/ALN NPs exhibit cytotoxic effects in vitro

**Fig. 4:**
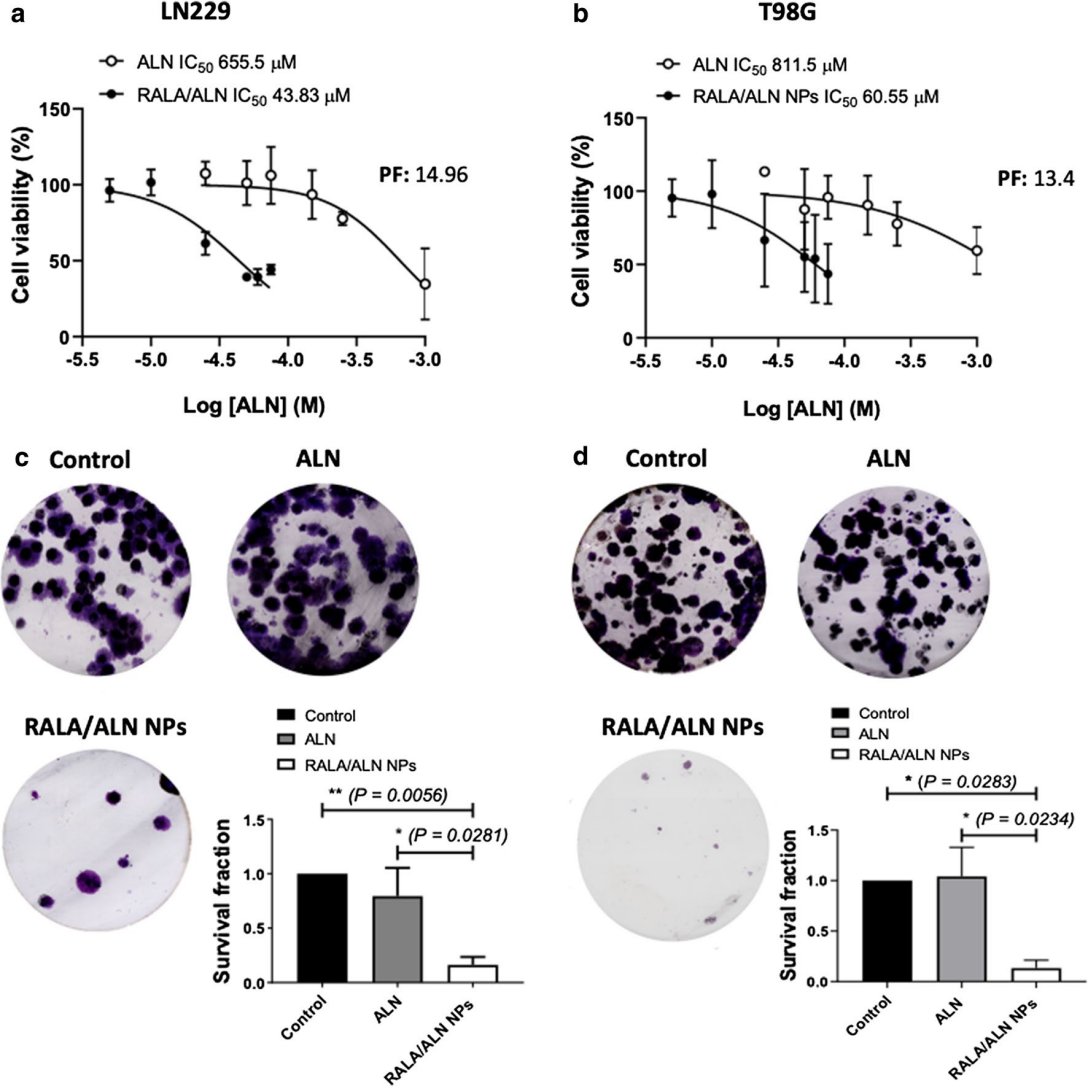
Dose-response curves and colony formation of cells treated with RALA/ALN NPs.** a** LN229 and **b** T98G glioblastoma cancer cells were treated with uncomplexed ALN to achieve a final concentration ranging from 5 μM to 1 mM and RALA/ALN NPs were prepared at a 10:1 mass ratio such that the final concentration of ALN per well ranged between 5 μM to 75 μM. Cells were treated for 6 h before medium was replaced with complete media and left to incubate for 72 h. Cell viability was assessed using MTS reagent and expressed as a percentage of the untreated control (considered to be 100% viable). The IC_50_ values were calculated using commercial software (GraphPad Prism® 8.1.2, GraphPad Software, USA). Data were obtained from three repeat MTS experiments. Clonogenic survival assay in c LN229 and d T98G cells treated with RALA/ALN NPs. Cells were treated for 6 h with uncomplexed ALN and RALA/ALN NPs prepared at mass ratio 10:1 to achieve a final ALN concentration of 43.83 μM and 60.55 μM in T98G and LN229 cell lines, respectively. The SF was calculated as the plating efficiency of the treated groups divided by the plating efficiency of untreated cells. Results are reported as mean ± SEM, n = 3

The cytotoxic effects of RALA/N-BP NP were determined using a MTS cell viability assay (Fig. 4a, b). Complexation with RALA augmented the potency of ALN in T98G cells by a potentiation factor of 13.4 at 72 h. The IC_50_ of uncomplexed ALN was 811.5 μM which reduced to 60 μM when treated with RALA/ALN NPs. The anticancer effects of RALA/ALN NPs were more pronounced in LN229 cells with the IC_50_ of uncomplexed ALN at 655.5 μM and 43.83 μM with the RALA/ALN NPs, a potentiation factor of 14.96.

Clonogenic assays were used to measure the long-term cytotoxic effects of RALA/ALN NPs. Cells were treated with the calculated IC_50_ concentration of RALA/ALN NPs, as determined by dose response curves in each cell line, (Fig. 4c, d). RALA/ALN NPs significantly inhibited long-term survival in LN229 and T98G cells. The most significant reduction in colony formation was observed in the LN229 cell line where the cytotoxic effects of ALN when complexed with RALA were observed (SF = 0.18) (*P<0.01*) compared to uncomplexed ALN and untreated controls (Fig. 4c). A similar effect was observed in T98G cells where RALA/ALN NPs significantly reduced cell survival compared to controls *(P<0.05)* (Fig. 4d).

### RALA/ALN NPs Induce apoptosis and inhibit the prenylation of small GTP-binding proteins in GBM cells

**Fig. 5:**
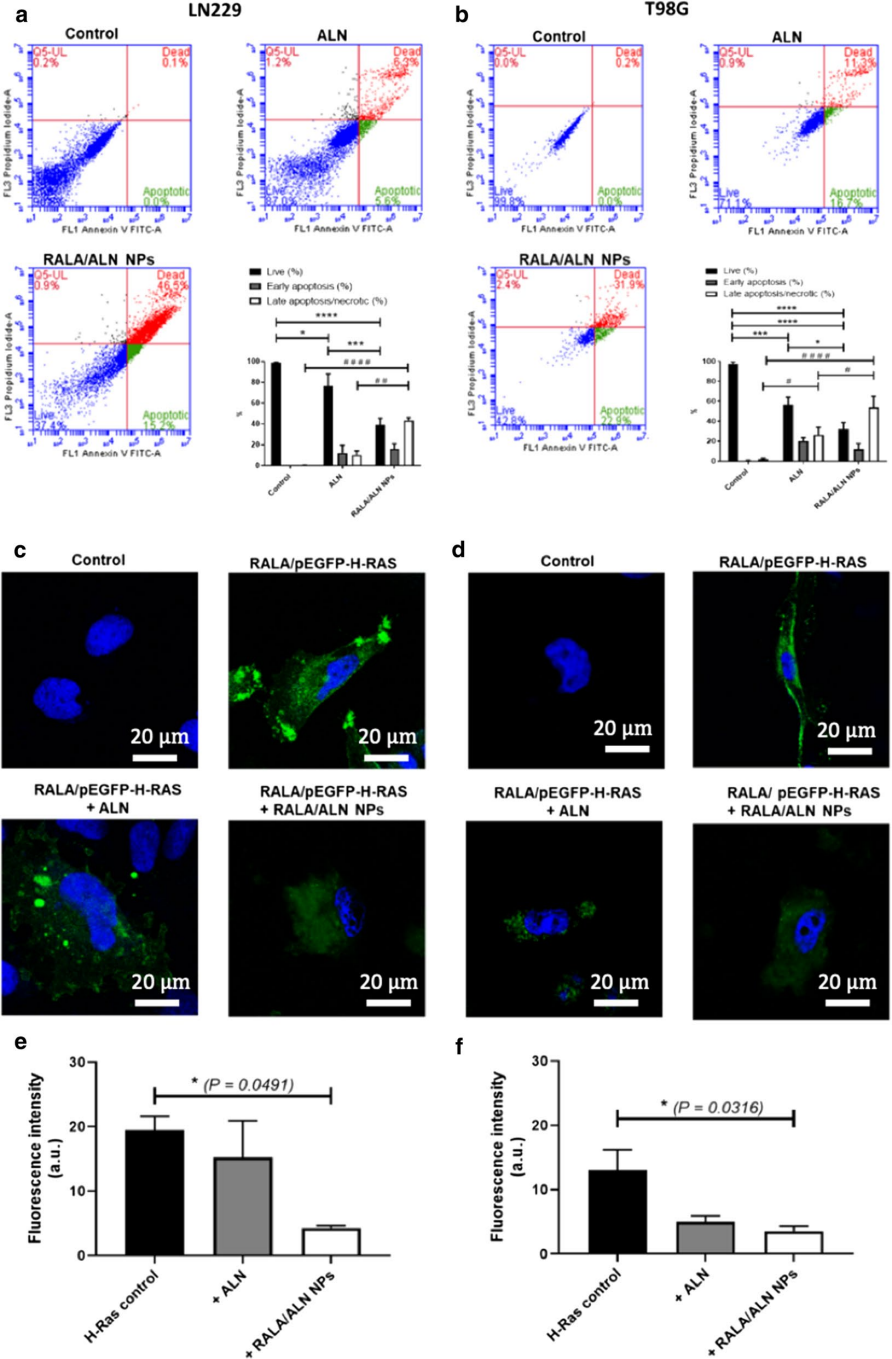
Flow cytometric analysis on the apoptotic effect of RALA/ALN NPs in **a** LN229 and **b** T98G cells. RALA/ALN NPs prepared at mass ratio 10:1, to achieve a final ALN concentration of 43.83 μM and 60.55 μM in T98G and LN229 cell lines, respectively. Cells were treated for 6 h before medium was removed and replaced with complete fresh media. Cells were left to incubate for 72 h prior to analysis. Cells were prepared for flow cytometry using the FITC Annexin V Apoptosis Detection Kit I (BD Biosciences Pharmingen™, USA) and PI staining as per manufacturers’ guidelines and analysed using the Accuri C6 Plus (BD Bioscience, UK) flow cytometer. Results are reported as mean ± SEM, n=3. Confocal microscopy images of H-Ras plasma membrane localisation in **c** LN229 and **d** T98G cells**.** Cells were transfected with 0.5 μg/μL plasmid EGFP-H-Ras for 4 h using the RALA peptide delivery system at N:P 10. After 24 h, cells were treated with either ALN or RALA/ALN NPs at a mass ratio of 10:1 (conc. 43.83 μM and 60.55 μM in T98G and LN229 cell lines, respectively.) for 6 h. Cells were incubated for 72 h prior to imaging using a TCS SP8-Leica Microsystems confocal microscope (Leica, UK) with a 63x oil objective lens and subsequently analysed using LAS AF Lite Software (Leica, UK). Images are representative of three independent repeats. Fluorescence quantification of plasma membrane localised H-Ras in **e** LN229 and **f** T98G cells. The green fluorescence in three independent confocal images was quantified using ImageJ (National Institute of Health, USA). Results are mean ± SEM, n = 3

The start of the apoptotic pathway is characterised by the translocation of phosphatidylserine (PS) from the inner leaflet to the outer membrane and as a result, enables binding of annexin-V to PS [30]. The annexin-V-FITC positive and PI negative labelled cells were considered as early apoptotic cells and were represented in green on the flow cytometric scattergrams (Fig 5a, b). Likewise, double positive annexin-V-FITC/PI cells were characterised as late apoptotic/necrotic and are evidenced as red on the flow cytometric scattergrams (Fig 5a, b). LN229 and T98G cells were treated with the calculated IC_50_ concentration of RALA/ALN NPs in each cell line, as determined by the dose response curves (Fig. 4a, b). The results in the RALA/ALN NP treatment group correlated to the IC_50_ dose response curves where similar proportions of viable and late apoptotic/necrotic cells were detected in the cell population (Fig. 5a, b). Uncomplexed ALN did not cause marked changes in viability compared to the untreated controls in the LN229 cell line. However, uncomplexed ALN decreased the percentage of viable cells in the T98G cell line to 56.6% 72 h post-treatment. Results demonstrate RALA complexed ALN significantly reduces cell viability and induces both early and late apoptosis in LN229 and T98G cancer cells. Furthermore, N-BPs are characterised as potent inhibitors of prenylation. To examine whether RALA complexation of ALN impacts the mode of action, the membrane localisation of GFP-H-Ras was studied as an indicator of prenylation. When GFP–H-Ras was transfected alone, or in conjunction with ALN in LN229 cells, plasma membrane localization was predominantly observed (Fig 5c, d). However, when co-treated with RALA/ALN NPs, GFP–H-Ras was absent from the plasma membrane and localisation was primarily in the cytosol or internal structures. ALN markedly decreased GFP-H-Ras prenylation in the T98G cell line as demonstrated by reduced green fluorescence on the outer membrane and concentrated regions of fluorescence within the cytosolic region. However, the green fluorescence on the plasma membrane decreased significantly further (*P *< 0.05) when cells were co-treated with RALA/ALN NPs.

### RALA/ALN NPs significantly inhibited GBM spheroid growth

**Fig. 6:**
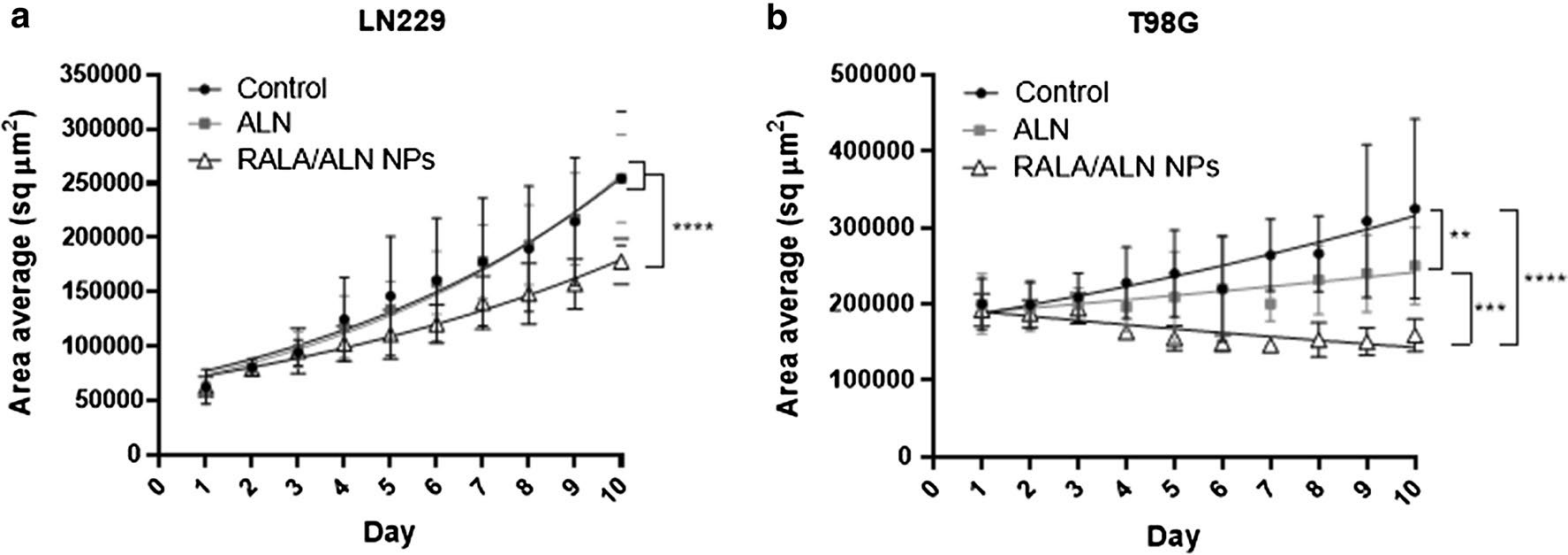
3D spheroid growth of RALA/ALN treated GBM cells.** a** LN229 and **b** T98G cells were treated with uncomplexed ALN and RALA/ALN NPs prepared at a 10:1 mass ratio, to achieve a final ALN concentration of 43.83 μM and 60.55 μM, respectively, in each well. Cells were incubated at 37 °C with 5% CO_2_ for 48 h to allow for spheroid formation. Spheroid measurement was initiated 72 h post-seeding and cells were treated on measurement Day 3. Spheres were measured daily over a 10-day period using the Cell3iMager X Neo (SCREEN, Shimadzu Japan) and maintained at 37 °C with 5% CO_2_ throughout. Results are displayed as mean ± SEM, n = 3

Although conventional 2D cell cultures cannot reproduce the complexity and heterogeneity of clinical tumours, [31] 3D models exhibit synonymous cell behaviour representative of *in vivo* conditions, providing more accurate biological results for *in vivo* translation. The objective of this study was to evaluate the effect of BPs on a spheroid 3D culture system using inhibition of growth as a measure of the anti-proliferative effects of RALA/ALN on LN229 and T98G cancer cells (Fig. 6). Spheroid growth was determined through area measurements obtained from the Cell3iMager X neo (SCREEN, Shimadzu Japan). RALA/ALN NPs significantly inhibited spheroid growth as evident in LN229 and T98G cells. Indeed, the spheroid area average started to decrease and plateau as soon as day 3 in the T98G cell line with the difference in area average highly significant at day 10 (*P *< 0.05) when compared with the untreated group. Untreated and ALN only treated LN229 spheroids grew significantly over the 10-day period, whereas when treated with RALA/ALN NPs, growth was reduced by 28% at Day 10 compared to untreated controls. Moreover, T98G spheroids treated with RALA/ALN NP exhibited complete inhibition of growth compared to untreated and ALN T98G spheroids (Table 1).

**Table 1:** Spheroid growth doubling time

	Doubling time (days)		
Cell line	Control	ALN	RALA/ALN
LN229	5.56 ± 0.74	5.18 ± 0.43	7.29 ± 0.36
T98G	14.00 ± 6.41	21.42 ± 8.30	–

### Lyophilisation of RALA/ALN NPs did not negatively impact NP formation or functionality

**Fig. 7:**
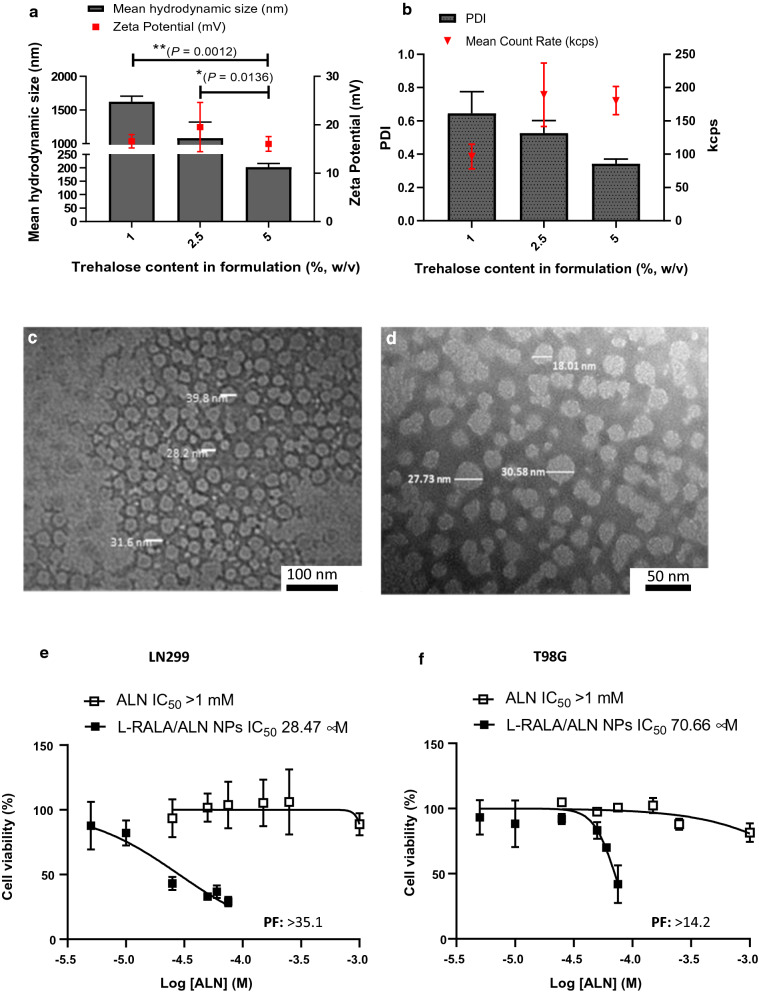
Lyophilisation of RALA/ALN NPs were prepared with a range of trehalose concentrations. The **a** mean hydrodynamic size and zeta potential of L-RALA/ALN NPs at mass ratio of 10:1 containing 1 μg ALN. Particles were made up to a 50 μL volume and left to incubate for 30 min at RT. Subsequently, particles were lyophilised to achieve a final trehalose concentration of 1%, 2.5% and 5% w/v before reconstitution in 50 μL of UltraPure DNase/RNase-Free Distilled Water. Particles were measured using the Malvern Zetasizer Nano ZS instrument at 20 °C through DLS and laser doppler electrophoresis, respectively. **b** The sample quality of L-RALA/ALN NPs was measured through corresponding PDI and the mean count rate (kcps). Results are displayed as mean ± SEM, n = 3. TEM images of **c** RALA/ALN NPs taken at 30 K magnification, and (D) L-RALA/ALN NPs were taken at 80 K magnification. NPs were loaded onto carbon reinforced 400 mesh copper grids (TAAB Laboratories, UK) which were subsequently stained with 5% uranyl acetate before drying. Grids were imaged using a JEM-1400Plus Transmission Electron Microscope (JEOL Inc., USA) at an accelerating voltage of 120 kV to provide high resolution images. (Scale bars = C: 100 nm, D: 50 nm). Cell viability of **e** LN229 and **f **T98G cells treated with L-RALA/ALN NPs as measured by MTS. Cells were treated with uncomplexed ALN to achieve a final concentration ranging from 5 μM to 1 mM and L-RALA/ALN NPs (1 μg ALN) were prepared at a 10:1 mass ratio such that the final concentration of ALN per well ranged between 5 μM to 75 μM. Lyophilised NPs were reconstituted in 50 μL of UltraPure DNase/RNase-Free Distilled Water prior to use. Cells were treated for 6 h before medium was replaced with complete media and left to incubate for 72 h. Post-incubation, cell viability was assessed using MTS reagent and cell viability was expressed as a percentage of the untreated control (considered to be 100% viable). The IC_50_ values were calculated using commercial software (GraphPad Prism®, 8.1.2, GraphPad Software, USA). Results are displayed as mean ± SEM, n = 3

RALA/ALN were lyophilised to enable increased drug loading and improve stability. L-RALA/ALN NPs were prepared at a mass ratio of 10:1 using trehalose as a cryoprotectant. The concentration of trehalose was varied to determine the final concentration required for lyophilisation without negatively impacting NP conformation (Fig. 7a, b). A trehalose concentration of 5% w/v was optimal to retain the physiochemical characteristics of RALA/ALN NPs post-lyophilisation. The mean hydrodynamic size of L-RALA/ALN NPs was 202 nm and particles retained a positive surface charge ~ 16 mV. A lower trehalose concentration at 1 and 2.5% w/v led to an increase in the mean hydrodynamic size to 1078.8 nm and 1621.33 nm, respectively, with a corresponding increase in PDI to 0.65 with 1% w/v trehalose compared to a PDI of 0.34 in the 5% w/v trehalose formulation. TEM analysis was used to visualise the structural characteristics of formulated RALA/ALN NPs and L-RALA/ALN NPs at a mass ratio 10 (Fig. 7c, d). Both formulated NPs presented a uniform distribution of spherical particles.

The functionality of RALA/ALN NPs was assessed after freeze-drying. GBM cells were treated with uncomplexed ALN or L-RALA/ALN NPs at mass ratio 10:1 over a range of concentrations for 6 h and then incubated for 72 h before measuring cell viability using MTS reagent. Dose-dependent cytotoxicity was elicited by uncomplexed ALN and RALA/ALN NPs after lyophilisation as shown in Fig. 7e, f. Similar to non-lyophilised NPs, L-RALA/ALN NPs potentiated the effects of ALN with the greatest anticancer effect observed in the LN229 cell line as demonstrated by an IC_50_ of 28.47 μM with a PF >35.1. (Fig. 7e. f; Table 2.). Furthermore, an IC_50_ values of 70.66 μM were calculated in T98G cells when treated with L-RALA/ALN NPs. Compared to non-lyophilised RALA/ALN NPs (Table 2; LN229–43.83 μM; and T98G–60.55 μM), the IC_50_ values obtained when cells were treated with L-RALA/ALN NPs resulted in a decrease of the IC_50_ in the LN229 cell line (Table 2).

**Table 2:** Summary table of the IC_50_ values of L-RALA/ALN NPs in LN229 and T98G cell lines 72 h post-treatment

	Fresh NPs	Lyophilised
Size	45.92 ± 2.69 nm	202.33 ± 13.47 nm
PDI	0.52 ± 0.069	0.34 ± 0.027

	IC_50_ (µM)			
Cell line	RALA/ALN	PF	L-RALA/ALN	PF
LN229	43.83	14.96	28.47	> 35.1
T98G	60.55	13.4	70.66	> 14.2

## Discussion

The work in this paper highlights how complexation of N-BPs with the RALA peptide delivery platform, augments the anticancer effects in GBM. LN229 and T98G cells treated with RALA/ALN NPs exhibited a significant decrease in the cell viability *in vitro*, as demonstrated by the apoptosis and clonogenic assay. These NPs are evidence of a promising novel therapeutic in the treatment of GBM.

The complexation of anionic compounds with the RALA peptide is dictated by electrostatic interactions. [17] BPs are distinguished by the unique ‘P-C-P’ structure where anionic phosphonate groups are at the core of the parent structure. [9] At pH 7.4, BPs such as ALN and risedronate (pKa 6.3 and 5.25, respectively) are deprotonated thereby facilitating electrostatic interactions with RALA. For example, Nancollas *et al.* found that at a higher pH (pH 7.4) more phosphonate protons dissociated to give rise to a negative BP species [32], which is advantageous for NP formulation; N-BPs were dissolved in TE buffer (pH 8.0) prior to NP formulation to increase the affinity for cationic RALA. ALN was stable in TE buffer over a 7-day period where the pH remained steady at pH 7–8. TE buffer was deemed biologically appropriate for formulation as it is commonly used for the preservation of DNA during storage, with no associated adverse effects observed in cells [33]. The composition of 1% Tris with synthetic amino acid EDTA maintained the solution at an alkaline pH. EDTA is a hexadentate ligand which sequesters ions such as Ca^2+^ and Mg^2+^. After being bound by EDTA into a metal complex, metal ions remain in solution but exhibit diminished reactivity [34]. Therefore it is possible that the inhibition of surrounding cationic ions within an aqueous solution strengthens the electrostatic interactions between RALA and N-BPs thus stabilising the peptide-NP complex, a method that could be employed for future formulations.

RALA/ALN NPs < 100 nm in size with an overall positive surface charge were formed with a zeta potential of + 16 mV or higher across all mass ratios. The PDI remained less than 0.7 with no significant difference observed at each individual mass ratio. Additionally, there was no significant change in particle size over a range of temperatures up to 40 °C and over 28 days, exhibiting the highly stable nature of the RALA/ALN NPs. Cationic NPs are advantageous for cell uptake, with particles binding strongly to lipid bilayers, triggering endocytosis. Indeed, Oh and Park found that positively charged cysteamine-gold NPs, up to 40 nm in size, were internalized at a higher rate compared to negatively charged or zwitterionic gold NPs in monocytes and macrophages. [35]

Given that ALN lacks a chromophoric group, AF647-RIS was used as a fluorescent N-BP analogue to quantify RALA complexation and cellular uptake of N-BPs. The presence of a bulky AlexaFluor®647 fluorescent group did not prevent RALA complexation. There was no significant increase in the mean hydrodynamic size as particles remained below 100 nm. However, the zeta potential significantly increased up to approx. + 24 mV. It is not possible to accurately predict how binding between the cationic peptide and varying moieties will occur due to the spontaneous nature of self-assembly, however, it is possible that due to the higher MW of AF647-RIS, additional RALA molecules concentrated at the particle surface for efficient complexation. It was shown that RALA effectively complexed fluorescently labelled AF647-RIS, providing an encapsulation efficiency > 95% at a mass ratio of 10:1, indicating highly efficient condensation.

Characteristically, BPs are taken up through fluid-phase endocytosis, which is an actin-dependent endocytic pathway where ruffling plasma membranes fuse to enclose fluid [5, 36, 37]. It is thought that during osteoclastic bone resorption, acidification of the vesicle leads to neutralisation of the phosphonate head groups, which in turn, facilitates diffusion across the endosomal membrane. However, this process results in the entrapment of the BP in the acidic interior of endosome vesicles so only a small amount is able to exert an effect within the cytosol [5]. Coxon *et al.* found the internalisation of N-BPs was higher in resorbing osteoclasts compared to non-resorbing osteoclasts, calvarial osteoblasts and MCF-7 breast cancer cells (those not associated with a resorption pit) [38]. Therefore, it is highly unlikely that BPs are able to be effectively taken up by non-resorbing, non-osseous cells to reach the concentrations required for full efficacy. RALA is particularly efficient in entering cells and escaping the endosome. RALA complexation could potentially reduce bone-targeting, leading to enhanced cellular uptake in non-osseous cell types through increased BP bioavailability. The uptake of AF647-RIS was observed across all mass ratios in both T98G and LN229 cell lines. Notably, the highest uptake was in LN229 cells which was confirmed through confocal microscopy. Inhibition of FPPS enzymatic activity occurs within the intracellular compartment and it is here BPs exert a therapeutic response.

Previous studies have proven RALA is capable of effectively escaping entrapment within the late endosome promoting delivery of anionic cargo to the site of action. [5, 16–22]

In this study RALA formulated BPs exert anticancer effects in a dose-dependent manner with both fresh and lyophilised NPs. A potentiation factor of 14.6 and 13.4 was achieved in both LN229 and T98G GBM cell lines, respectively, when cells were treated with RALA/ALN NPs compared to free drug alone. Dose response studies showed particles retained functionality post-lyophilisation where the cytotoxicity of ALN was potentiated through RALA complexation and notably demonstrated more potent effects on cell viability. The greatest potentiation was observed in LN229 cells compared to T98G cells at a factor of > 35.1 and > 14.2, respectively, when treated with lyophilised NPs. Lyophilisation is essential if these NPs were to become a product as electrostatic NPs are destabilised in saline solution due to the presence of NaCl molecules. Sugars such as trehalose are advantageous as they do not impact upon charge and, in fact, create a product that upon re-constitution becomes isotonic. The optimal trehalose concentration was determined with DLS and TEM images indicating that particles retained a uniform spherical nanosized structure post-lyophilisation and notably lead to a lower PDI. Although the process of freeze-drying aids in establishing formulation stability, particle size increased post-lyophilisation which may have impacted cellular uptake of RALA/ALN NPs. Seyfoddin *et al.* found the particle size of solid lipid NP formulations significantly increased post-freeze drying [39]. Authors observed this was due to aggregation and suggested the addition of an effective cryoprotectant such as 5–10% trehalose, sucrose, or mannitol for better results. Although lyophilisation of RALA/ALN NPs in the presence of 5% trehalose minimised aggregation to provide 200 nm particles, further studies incorporating trehalose concentrations higher than 5% w/v may be useful to determine the optimal trehalose concentration which effectively protects NPs and does not negatively impact particle size or functionality.

Additionally, current evidence supports endocytosis as the mechanism utilised by cationic nanocomplexes for cell entry. McCarthy *et al.* demonstrated RALA NPs utilise clathrin and caveolae mediated endocytosis. [17] These cell entry pathways have been reported to mediate the entry of particle up to 200 nm in size. [40] Post-lyophilisation a particle mean diameter of 202.33 nm was observed. As a number of particles surpassed the threshold required for endocytic uptake, other uptake mechanisms may occur in addition to receptor-mediated endocytosis, namely, phagocytosis (particle size: 0.5–10 μm) and macropinocytosis (particle size: 100 nm–5 μm). [41, 42] We hypothesise that the increased uptake of L-RALA/ALN NPs maybe due to these additional uptake pathways leading to greater intracellular accumulation of ALN with increased cytotoxicity.

Dual staining with PI and FITC-Annexin V was used to evaluate the apoptotic effects of RALA/ALN NPs on LN229 and T98G cells. Results were aligned with the IC_50_ dose after 72 h when cells exhibited close to equal populations of live and late apoptotic/necrotic cells. Differences in apoptosis could be attributed to gene expression between cell lines. Hong *et al.* reported that LN229 and U251n GBM cells expressed multiple stem cell markers such as Nestin, Sox2, Musashi-1 and CD44 with evidence of higher migration and colony formation potential compared to T98G and U87 cells, which did not express Nestin, Sox2 and Musashi-1 [43]. Furthermore, the fast-growing nature demonstrated by LN229 cells, could correlate to a higher sensitivity to cytotoxic therapies. For example, Wang *et al.* evaluated the expression levels of selected markers in a panel of GBM cell lines and determined the sensitivities of GBM cell lines in response to TMZ. The authors found U87 and LN229 were more sensitive to TMZ treatment than T98G and LN18 and additionally, the expression levels of the genes ABCC3, TNFRSF1A, and MGMT were higher in T98G and LN18 than those in U87 and LN229. Notably ABCC3 is part of the ABC subfamily of multidrug resistance proteins, known to be a major detriment to cancer therapy [38, 44]. ABCC3 is an organic anion transporter which facilitates the efflux of a range of conjugated and unconjugated organic anions, as well as a range of therapeutic agents such methotrexate [45]. Therefore, it is possible that efflux of the negatively charged ALN, could be modulated by ABCC3 in T98G cells, resulting in reduced potency of RALA/ALN NPs in this particular cell line. Further studies ABCC3 knockdown studies could confirm this.

RALA/ALN NPs proved effective in the inhibition of cell growth, cell survival and induction of apoptosis *in vitro*. However, it was necessary to determine the mechanism by which this occurred. For example, Ras mutations are found in approximately 30% of all cancers with some cancers having much higher mutation rates [46]. These mutations are a negative predictor for a number of therapies, therefore disruption of Ras membrane localisation presents a potential anticancer strategy [47]. These findings indicate that H-Ras prenylation was significantly inhibited in all cell lines when treated with RALA/ALN NPs, indicating one likely mode of action. In a similar study, Lühe *et al.* reported an inhibition of FPPS occurred 1 h after N-BP treatment *in vitro* followed by decreased levels of prenylated proteins at 24–48 h and increased cytotoxicity post 48 h, findings comparable to ours [48]. This suggests protein prenylation must be reduced below a critical level before the cytotoxic effects occur typically 72 h post-treatment. Indeed, abnormal protein prenylation has been attributed in the progression of numerous cancer types including prenylation of Ras and Rho which has been implicated in gliomas [49]. However, it would be prudent to conduct further studies where other small GTPase variants are utilised to elucidate the full extent of the anticancer effects of RALA/ALN NPs in GBM. Furthermore, application of RALA/ALN NPs could be broadened to include other cancer types where Ras mutations are predominant (i.e. pancreatic cancer) [50].

Subsequently, spheroid models were established to simulate solid tumour conditions *in vitro* using RALA NPs for the first time. Impressively, a significant decrease in spheroid growth and doubling time was observed upon treatment with RALA/ALN NPs in both LN229 and T98G cell lines, highlighting the potential antitumour effects *in vivo.* Lagies *et al.* found that 3D spheroid cultures shared strikingly metabolic similarities to the organ tissue such that authors suggested optimised 3D culture techniques could possibly replace animal testing [51]. Furthermore, Di Liello *et al.* cultured 3D spheroids from patient derived non-small cell lung cancer tumours for *ex-vivo* analysis [52]. Authors reported that drug sensitivities to cisplatin-based chemotherapy and anti-programmed death 1 drug sensitivity were consistent from the patient clinical response to 3D culture. The results highlight how 3D cultures enable the formulation of highly translatable therapies for clinical use and demonstrate RALA/ALN NPs could significantly reduce tumour burden *in vivo*.

Currently, BPs do not reach significant accumulation levels in the brain after intravenous administration due to a high predisposition to distribute in skeletal regions [53]. Systemic administration of BP would require the use of a high quantities, exceeding the clinical dosing regimens given to patients to achieve an effective concentration in the brain. Therefore, delivery of BPs complexed with the RALA peptide holds clinical relevance as it could lead to a reduction in the therapeutic dose required as indicated by the significant potentiation effect. Taken together, this study indicates that the RALA/ALN NPs could be a nano-formulation to deliver ALN, an FDA-approved prenylation inhibitor, into GBM tumour tissues for enhanced anti-tumour effects.

## Conclusion

Reformulation strategies have been applied to N-BPs to reposition BPs as anticancer therapeutics. In the present study, we showed that an ALN-NP formulation prepared using the RALA peptide delivery platform enhanced the intracellular delivery of ALN to promote cell apoptosis, inhibit cell growth and prenylation *in vitro*. Although, many hurdles must be overcome before the full potential of non-viral, peptide-based delivery systems can be clinically translated, this data set indicates an encouraging pre-clinical data set.
